# Notes and Notices

**Published:** 1898-01

**Authors:** 


### NOTES AND NOTICES.

THE INTERNATIONAL MEDICAL ANNUAL for 1898, a work of reference for medical practitioners, is a continuation of the same work that has been issued regularly year after year by E. B. Treat & Co., 241, 243 W. 23d St., New York, and has been reviewed in these pages, and is, therefore, familiar to our readers. The issue for this year is rapidly approaching completion, and will prove the handiest, best arranged, and best edited reference volume issued to the medical profession. Among the special articles will be one on "The Chief Pathogenic Bacteria in the Human Subject," by S. G. Shattock, F. R. C. S. It will be illustrated by ten finely colored plates. There will also be two remarkable articles on "The Obliteration of the Deformity in Pott's Disease," by Dr. Robert Jones and Dr. A. H. Tubby. When issued a review will be published by us, giving the reader adequate information upon its merits.

